# National-Level Disparities in Internet Access Among Low-Income and Black and Hispanic Youth: Current Population Survey

**DOI:** 10.2196/27723

**Published:** 2021-10-12

**Authors:** M Margaret Dolcini, Jesse A Canchola, Joseph A Catania, Marissa M Song Mayeda, Erin L Dietz, Coral Cotto-Negrón, Vasudha Narayanan

**Affiliations:** 1 Hallie Ford Center for Healthy Children and Families College of Public Health and Human Sciences Oregon State University Corvallis, OR United States; 2 StatCon Consulting Hayward, CA United States; 3 Acumen LLC Burlingame, CA United States

**Keywords:** internet access, smartphone use, Black youth, Hispanic youth, low-income youth, disparities, mobile phone

## Abstract

**Background:**

Internet access is increasingly critical for adolescents with regard to obtaining health information and resources, participating in web-based health promotion, and communicating with health practitioners. However, past work demonstrates that access is not uniform among youth in the United States, with lower access found among groups with higher health-related needs. Population-level data yield important insights about access and internet use in the United States.

**Objective:**

The aim of this study is to examine internet access and mode of access by social class and race and ethnicity among youth (aged 14-17 years) in the United States.

**Methods:**

Using the Current Population Survey, we examined internet access, cell phone or smartphone access, and modes of connecting to the internet for adolescents in 2015 (unweighted N=6950; expanded weights N=17,103,547) and 2017 (unweighted N=6761; expanded weights N=17,379,728).

**Results:**

Internet access increased from 2015 to 2017, but socioeconomic status (SES) and racial and ethnic disparities remained. In 2017, the greatest disparities were found for youth in low-income households (no home access=23%) and for Black youth (no home access=18%) and Hispanic youth (no home access=14%). Low-income Black and Hispanic youth were the most likely to lack home internet access (no home access, low SES Black youth=29%; low SES Hispanic youth=21%). The mode of access (eg, from home and smartphone) and smartphone-only analyses also revealed disparities.

**Conclusions:**

Without internet access, web-based dissemination of information, health promotion, and health care will not reach a significant segment of youth. Currently, SES and racial and ethnic disparities in access prolong health inequalities. Moreover, the economic impact of COVID-19 on Black, Hispanic, and low-income communities may lead to losses in internet access for youth that will further exacerbate disparities.

## Introduction

Access to the internet is increasingly critical for adolescents with regard to health, employment, and education [1-7]. Furthermore, the COVID-19 pandemic has highlighted the fundamental importance of web-based access when in-person channels of communication are blocked [2,8]. Youth may use the internet to find health-related information, find resources, or participate in web-based interventions addressing a variety of physical and mental health topics [3,9-11] and for communicating with health providers [12,13]. Given that health-related conditions (eg, obesity, diabetes, and sexually transmitted infections) disproportionately impact low-income and racial and ethnic minority youth [14-16], access may be particularly important for these groups. Those with intermittent, limited, or no access to the internet will be disadvantaged because their ability to benefit from web-based health-related resources and programs, job searches, and educational activities will be lower than among those who have stable internet access [17-21].

Rapid increases in internet access have been attained over the past decade; however, US national household data from the American Community Survey (ACS) and the Current Population Survey (CPS) have demonstrated that we have yet to achieve the goal of universal access to the internet [22]. Furthermore, they also document continued disparities in access for Black and Hispanic people, low-income households, and those in rural areas [23]. Although the CPS and ACS found that internet access is higher in households with members ≤18 years of age, published analyses of national probability household surveys infrequently focus on internet access for adolescents.

Valuable data on internet access among adolescents can be obtained from probability-based national surveys conducted by the US Census, including the CPS. Prior analyses of the CPS (2012 data) showed that youth aged 14-17 years living in low-income households had lower internet access than youth in moderate- and high-income households (household income <US $25,000=63%, US $25,000-$49,999=80%, and ≥US $50,000=95%) [4]. In addition, Black and Hispanic youth had lower in-home access than Asian and White youth (White=89%, Black=73%, Asian=93%, and Hispanic=76%). This research, which focused primarily on Black youth, also revealed disparities within racial and ethnic groups. That is, Black youth living in low-income households had lower access than Black youth living in households with middle or high incomes (household income <US $25,000=52%, US $25,000-$49,999=77%, and ≥US $50,000=93%) [4]. Cell phone access was lowest among both low- and high-income Black youth. Furthermore, Black youth have limited access to the internet in the community (schools=60%, libraries=27%, and community centers=5%), highlighting the importance of home access [4]. Given the broadscale uptick in internet access observed in the population at large, we anticipate that youth access will also have increased in recent years.

Other national studies on youth suggest substantial increases in access over time and a more even distribution of internet access across subgroups of youth [1,24,25]. A recent Pew Research Center report showed nearly universal internet access among youth in the United States (99%), with 95% reporting access to a smartphone (White=94%, Black=94%, and Hispanic=95%). Furthermore, relatively small differences in access by household income were reported (<US $30,000=93%, US $30,000-$74,999=93%, and ≥US $75,000=97%) [1]. However, these point estimates may conceal true differences. When CIs are taken into consideration, the Pew Research Center study shows that access for Black youth may range from 82.1% to 100%, and for Hispanic youth, the estimated range is 85.5%-100% (ie, margin of error by race and ethnicity: White=–7.2 to +7.2 points, Black=–11.9 to +11.9 points, and Hispanic=–9.5 to + 9.5 points). Similarly, CIs for income categories are wide (ie, margin of error by income: <US $30,000=–9.6 to +9.6 points, US $30,000-$74,999=–8.3 to +8.3 points, and ≥US $75,000=–8.1 to +8.1 points), suggesting that access by income may also be more variable. In light of this, it is critical that other sources, such as census data (eg, CPS and population studies with reduced error) are used to provide a sharper picture of access [22].

This research examines changes in access and use of the internet among those aged 14-17 years using the 2015 and 2017 CPS. We focus specifically on 2 racial and ethnic groups that have previously had lower access, Black and Hispanic youth, to determine whether disparities in access for racial and ethnic minorities and low-income youth have decreased over time. In light of recent research on adults, which suggests that the number of smartphone-only internet users in the United States is increasing [26], we also examined smartphone-only use among youth.

## Methods

### Data Source: The CPS

The CPS periodically collects data on computer and internet use, which provides national-level estimates of these topics. This household probability study conducted by the US Census Bureau is the primary source of labor force statistics [27,28]. Data for this study were obtained from the CPS Computer and Internet Use supplement conducted in July 2015 and November 2017. The supplement is sponsored by the National Telecommunications and Information Administration.

We used the 2 most recent surveys for which data are available to examine internet use inside and outside the home and additional modes (eg, devices) used to access the internet. Given our focus on youth, we limited our examination to household members aged 14-17 years in 2015 (total sample unweighted: N=6950; Black: n=917; Hispanic: n=1412) and 2017 (total sample unweighted: N=6761; Black: n=800; Hispanic: n=1377). This age group represents minors most likely to be in high school and living at home and corresponds to the age range examined in prior analyses of CPS 2012 data [4].

### Measures and Analyses

All analyses were conducted for a subsample of the CPS representing youth aged 14-17 years. Data on youth were collected from an adolescent aged ≥15 years or from a proxy. We examined four primary questions (yes or no responses) that were assessed in 2015 and 2017. Three items were identical in both waves: “Do you/Does anyone in this household, including you, use the internet at home?” “Who uses the internet at home?” “Who uses a cellular phone or smartphone?” The item regarding smartphone use was asked slightly differently across the two waves (2015: “Do you/Does anyone in this household use a cellular phone or smartphone?” 2017: “What about a smartphone or a cell phone that connects to the internet? Do you/Does anyone in this household use a smartphone?”). In addition, we constructed a variable to identify smartphone-only use, which was defined as accessing the internet from smartphone but not accessing the internet from home.

Analyses were conducted for all racial and ethnic groups, followed by analyses for Black and Hispanic youth separately. Cross tabulations are weighted using the final person weight [29]. Data analyses were generated using SAS software, version 9.3 of the SAS System (SAS Institute Inc; SAS and all other SAS Institute Inc product or service names are registered trademarks or trademarks of SAS Institute Inc). Standard survey weights adjusted with expansion weights provide estimates of the size of the population to which data are generalized (expanded weights for total sample: N=17,103,547 [2015] and 17,379,728 [2017]; Black: N=2,626,139 [2015] and 2,560,101 [2017]; Hispanic: N=3,867,713 [2015] and 3,981,899 [2017]). Owing to the population sizes, the CIs around the point estimates are very tight (ie, for proportions, –1% to +1% on either side) and can be interpreted as population parameters. For illustration purposes, we calculated the 95% CI for access by income for Black households with those aged 14-17 years and found that the CIs for all 3 categories of income were less than 1% (eg, <US $25,000=27.37% (95% CI 27.32-27.42), US $25,000-$50,000=27.7% (95% CI 27.65-27.67), and >US $50,000=44.93% (95% CI 44.87-44.99). Finally, we do not conduct statistical hypothesis testing for differences between groups because very large population sizes will invariably produce statistically significant differences [30-32].

## Results

### Overview

The results examine 2 major elements: internet access and the mode of internet access. To report on internet access, we examined the proportion of youth residing in households that have internet connection and the proportion of youth residing in households in which an adolescent, aged 14-17 years, has a cell phone or smartphone. The mode of internet access is reflected in data on (1) the proportion of adolescents accessing the internet through home internet connections, (2) cell phone or smartphone internet connections, and (3) those who have accessed the internet only through smartphones (ie, smartphone only). We examined internet access and mode of internet access for 2015 and 2017 with a focus on racial and ethnic minority and social class differences for the population as a whole and social class differences within Black and Hispanic populations.

**Figure 1:**
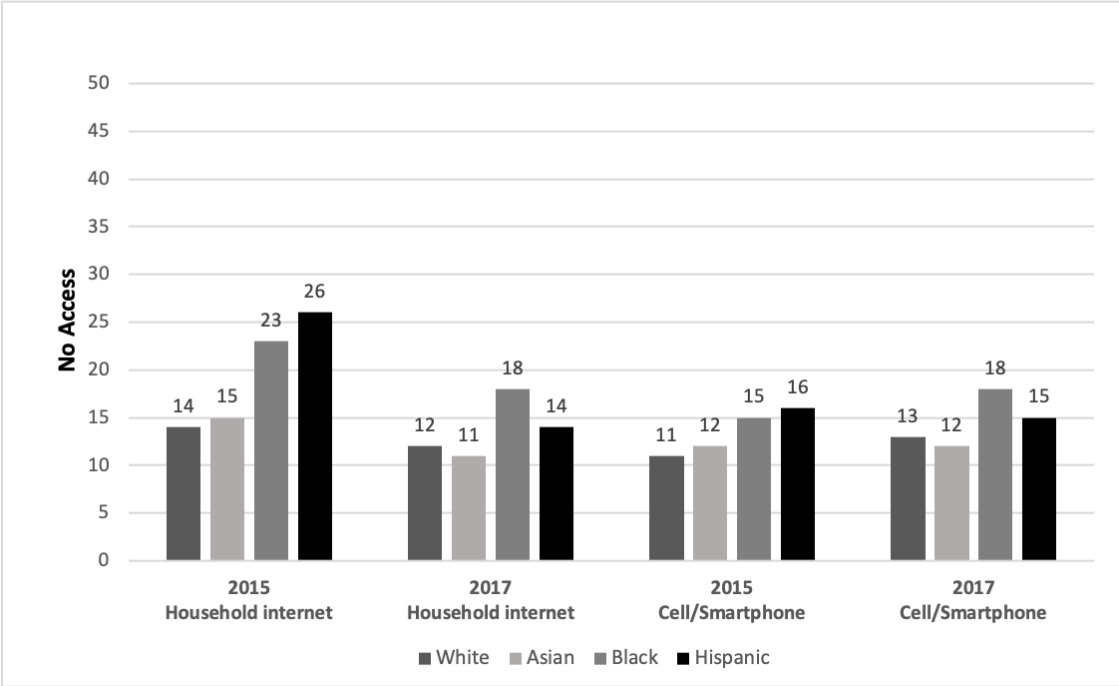
Disparities in access by race and ethnicity 2015-2017. The figure shows the percentage of youth (aged 14-17 years) who do not have access; household internet: internet access in the home; cell phone/smartphone: has cell phone or smartphone.

**Figure 2:**
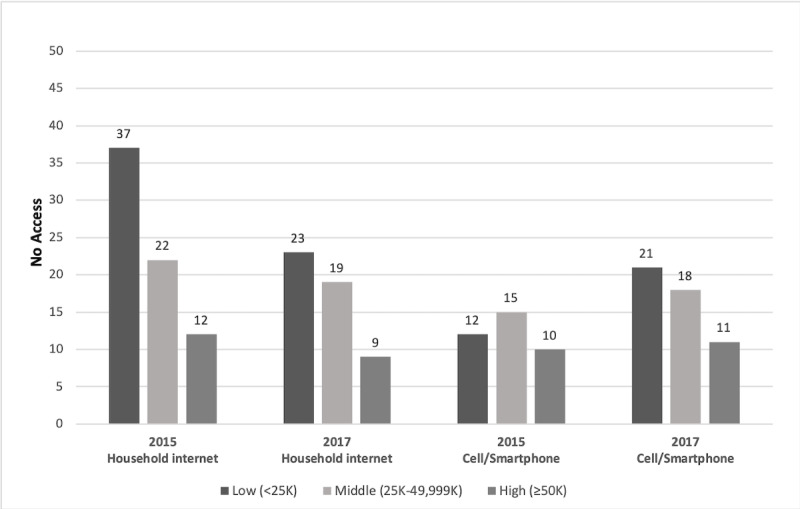
Disparities in access by income 2015-2017. The figure shows the percentage of youth (aged 14-17 years) who do not have access; household internet: internet access in the home; cell phone/smartphone: has cell phone or smartphone.

**Figure 3:**
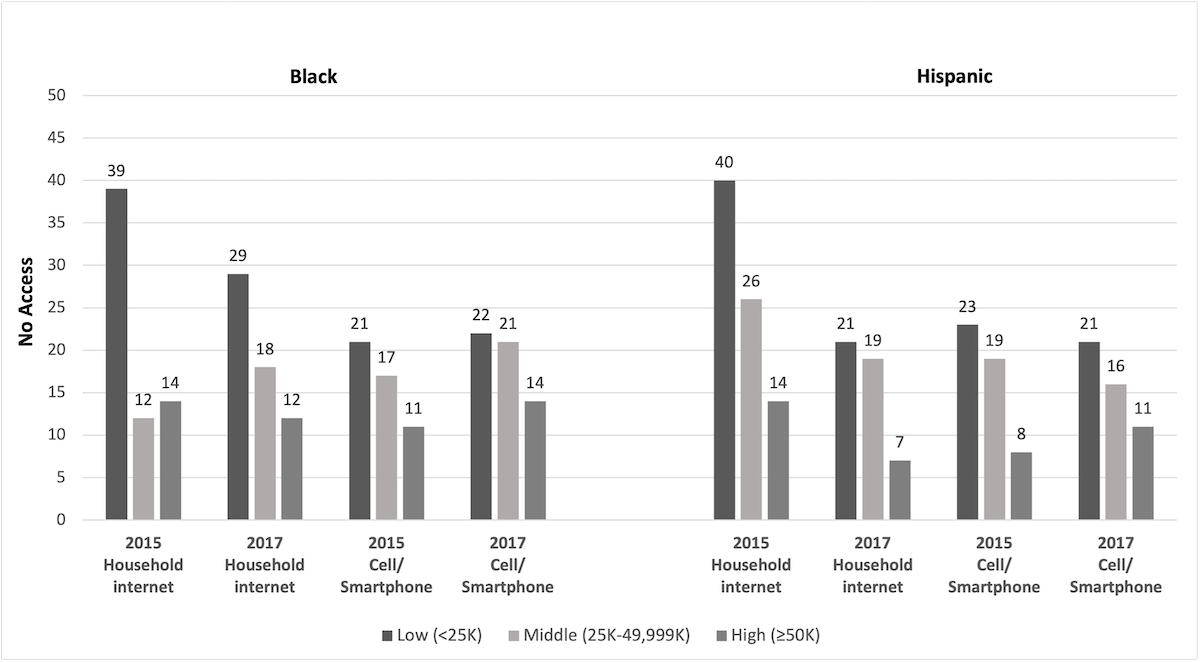
Disparities in access by income among Black and Hispanic youth. The figure shows the percentage of youth (aged 14-17 years) who do not have access; household internet: internet access in the home; cell phone/smartphone: has cell phone or smartphone.

Table 1 presents the percentage of those aged 14-17 years (1) living in a household with internet, (2) who have a cell phone or smartphone, and (3) with modes of accessing the internet (eg, home and smartphone) for 2015 and 2017 by racial and ethnic group and household income for the total population of households with an adolescent resident. Table 2 presents data separately by household income for Black and Hispanic youth, the 2 racial and ethnic groups that evidence the lowest access. In the text, we highlight the disparities in access and use by discussing the proportion of youth who lack access overall and through specific modalities (Figures 1-3 present data on lack of access; parallel data on modalities are not included in figures). Our disparity analysis assumes the ideal goal of universal internet access (ie, 100%).

**Table 1:** Reported internet use in the United States: youth aged 14-17 years by selected characteristics (Current Population Survey 2015 and 2017)^a^.

Characteristic		Individual lives in household with internet use^b^, n (%)			Individual uses a cell phone or smartphone, n (%)			Mode of internet access, n (%)				
		Year, 2015	Year, 2017	Year, 2015		Year, 2017	Home				Smartphone	
							Year, 2015		Year, 2017	Year, 2015		Year, 2017
**Race and Hispanic origin^c^**												
	White non-Hispanic	7.9 (86)	8.2 (88)	8.2 (89)		8.1 (87)	7.3 (79)		7.3 (78)	6.5 (70)		6.5 (70)
	Black	2.0 (77)	2.1 (82)	2.2 (85)		2.1 (82)	1.7 (67)		1.8 (70)	1.6 (61)		1.5 (59)
	Asian	0.7 (85)	0.8 (89)	0.8 (88)		0.8 (88)	0.6 (75)		0.7 (78)	0.7 (68)		0.6 (71)
	Hispanic (of any race)	2.9 (74)	3.4 (86)	3.2 (84)		3.4 (85)	2.5 (66)		3.0 (75)	2.3 (59)		2.5 (63)
**Household income^c^ (US $)**												
	<25,000	1.9 (63)	2.1 (77)	2.4 (78)		2.2 (79)	1.6 (52)		1.8 (66)	1.5 (49)		1.6 (56)
	25,000-49,999	3.0 (78)	3.0 (81)	3.3 (85)		3.1 (82)	2.7 (70)		2.6 (71)	2.4 (62)		2.3 (61)
	≥50,000	9.0 (88)	9.9 (91)	9.2 (90)		9.7 (89)	8.3 (81)		8.8 (80)	7.4 (73)		7.8 (72)

^a^Data derived from the CPS 2015 [33] and 2017 [34]. Percentages are rounded to whole numbers. Counts are presented in the millions and rounded to the nearest tenth of a million or nearest one-hundredth of a million if less than 1 million. Thus, 7.9 represents 7.9 million.

^b^At least 1 member of the individual’s household reported using the internet from home, even if that individual did not report use themselves.

^c^Data collected from respondents aged 14-17 years. Standard survey weights were adjusted with expansion weights to estimate the size of the population to which the data are generalized. Expanded weights N=17,103,547 (2015) and N=17,379,728 (2017). Unweighted N=6950 (2015) and N=6761 (2017).

**Table 2:** Reported internet usage in the United States by income: Black and Hispanic youth aged 14-17 years by selected characteristics (Current Population Survey 2015 and 2017)^a^.

Race and household income				Individual lives in household with internet use^b^, n (%)				Individual uses a cell phone or smart phone, n (%)					Mode of internet access, n (%)							
				Year, 2015		Year, 2017		Year, 2015		Year, 2017		Home						Smartphone		
												Year, 2015			Year, 2017		Year, 2015			Year, 2017
**Black^c^**																				
	**Household income^c^ (US $)**																			
		<25,000	0.4 (61)		0.5 (71)		0.6 (79)		0.5 (78)		0.3 (48)			0.4 (61)		0.3 (47)			0.4 (57)	
		25,000-49,999	0.5 (78)		0.6 (82)		0.5 (83)		0.6 (79)		0.5 (70)			0.5 (66)		0.4 (66)			0.4 (54)	
		≥50,000	1.1 (86)		1.0 (88)		1.1 (89)		1.0 (86)		0.9 (76)			0.9 (78)		0.8 (67)			0.7 (65)	
**Hispanic origin^c^**																				
	**Household income^c^ (US $)**																			
		≤25,000	0.7 (60)		0.8 (79)		0.9 (77)		0.8 (79)		0.6 (52)			0.7 (68)		0.6 (48)			0.5 (54)	
		25,000-49,999	0.9 (74)		1.0 (81)		1.0 (81)		1.0 (84)		0.8 (66)			0.9 (72)		0.7 (57)			0.7 (61)	
		≥50,000	1.2 (86)		1.7 (93)		1.3 (92)		1.6 (89)		1.1 (77)			1.4 (80)		1.0 (70)			1.2 (69)	

^a^Data derived from the CPS 2015 [33] and 2017 [34]. Percentages are rounded to whole numbers. Counts are presented in the millions and rounded to the nearest tenth of a million or nearest one-hundredth of a million if less than 1 million. Thus, 2.0 represents 2.0 million.

^b^At least one member of the individual’s household reported using the internet from home, even if that individual did not report use themselves.

^c^Data collected from respondents aged 14-17 years. Standard survey weights were adjusted with expansion weights to provide an estimate of the size of the population to which the data are generalized. Black: expanded weights, N=2,626,139 (2015) and N=2,560,101 (2017). Unweighted N=917 (2015) and N=800 (2017). Hispanic: expanded weights N=3,867,713 (2015) and N=3,981,899 (2017). Unweighted N=1412 (2015) and N=1377 (2017).

### Disparities in Internet and Cell Phone or Smartphone Access 2015-2017

#### Youth Access: Population Profile

In 2015, no group had 100% access, and racial, ethnic, and income disparities were evident (Figures 1 and 2). Up to one-fourth of youth lived in households without internet access; Black and Hispanic youth evidenced the greatest disparities. Overall, low-income youth were most likely to have no home internet access. Similarly, more Black and Hispanic youth and youth in middle-income households reported not using cell phones or smartphones. In 2017, there continued to be disparities despite gains in household internet access from 2015 to 2017 (Table 1). Although disparities by income and across racial and ethnic groups decreased in 2017 (Figures 1 and 2), low-income and Black and Hispanic youth continued to lag behind other groups. In some groups, fewer youth reported having cell phones or smartphones in 2017, revealing small increases in disparities overall from 2015 to 2017. Racial and ethnic disparities in cell phone or smartphone use in 2017 remained (Figure 1).

#### Youth Access: Disparities Among Black and Hispanic Youth

Socioeconomic status differences were evident among Black and Hispanic youth in 2015 (Figure 3). Nearly 3 times as many low-income compared with high-income Black and Hispanic youth lacked home access to the internet in 2015. Approximately 40% of low-income Black and Hispanic youth did not have home internet access. Cell phone and smartphone use followed a similar pattern, with greater disparities for low-income youth. In 2017, Black and Hispanic youth in the lowest-income households continued to evidence the greatest disparities. A total of 29% and 21% of low-income Black and Hispanic youth, respectively, lacked home access. In contrast, 12% and 7% of high-income Black and Hispanic youth, respectively, lacked access. Cell phone and smartphone use followed similar patterns (Figure 3).

### Mode of Internet Access 2015-2017

#### Youth’s Mode of Access: Population Profile

We examined modes of youth use for connecting to the internet, including accessing the internet from home or from a smartphone (Table 1). In 2015, more than 20% of youth did not access the internet from home, and at least 30% did not access the internet from a smartphone; Black and Hispanic youth were less likely than other groups to access from either mode. Overall, youth in low-income households were least likely to access the internet from home or from a smartphone in 2015; gaps between the lowest and highest income households were substantial. In 2017, a large proportion of youth still did not access the internet from home. Some racial and ethnic disparities observed in 2015 were reduced because more Hispanic youth reported accessing the internet from home. Disparities in the use of smartphones to access the internet also remained in 2017. Overall, nearly one-third of the youth did not access the internet from a smartphone (population average=33%). An income gap in accessing the internet from home continued to exist in 2017, although a substantial increase in access among low-income households narrowed the gap. Surprisingly, there were only modest changes in accessing the internet from smartphones between 2015 and 2017, with lower use reported overall. Youth in low-income households continue to be the most likely to not access the internet from a smartphone.

#### Mode of Access: Disparities Among Black and Hispanic Youth

Table 2 includes the mode of access data for Black and Hispanic youth. In 2015, Black and Hispanic youth in the lowest-income households were substantially more likely than those in households with higher income to lack access to the internet from home. Strikingly, about half of low-income Black and Hispanic youth did not access the internet from home or from a smartphone. Income disparities in access modality decreased from 2015 to 2017, with more low-income Black and Hispanic youth reporting access to the internet from home or smartphones in 2017. Despite this change, the disparity between low- and upper-income brackets continues to be evident in 2017. More specifically, 39% and 32% of low-income Black and Hispanic youth, respectively, did not access the internet from home in 2017. Similarly, 43% and 46% of low-income Black and Hispanic youth, respectively, did not access the internet from a smartphone in 2017.

#### Mode of Access: Smartphone Only

We examined whether the youth accessed the internet *only* from a smartphone for both time points (eg, smartphone-only users; data not presented). In 2015, nearly 10% of the youth were smartphone-only users, with Hispanic youth having the largest percentage with smartphone-only access (White=5%, Asian=7%, Black=8%, and Hispanic=9%). In addition, youth in low-income households were the most likely to have only smartphone access (12%); this was also true among Black and Hispanic youth. In 2017, there was a small reduction in reports of smartphone-only access; Hispanic youth were again the most likely to report only smartphone access (White=3%, Asian=3%, Black=3%, and Hispanic=5%). Youth in low-income households continued to be the most likely to have smartphone-only access in 2017 (6%). Among Blacks, low-income youth were more likely to report smartphone-only access in 2017, whereas among Hispanics, youth in middle-income households were the most likely to report smartphone-only use.

## Discussion

### Principal Findings

Using the CPS, we found that internet access has not reached universal levels, despite the critical role that the internet plays in connecting youth to health information, interventions, and providers. On a positive note, we observed a reduction in disparities from 2015 to 2017, with the largest gains typically found among the racial, ethnic, and income groups that lagged behind. Despite these gains, Hispanic and Black youth continue to have lower levels of household access. Regardless of racial or ethnic group membership, youth in low-income households remain at a disadvantage. Even with recent gains, nearly 30% of low-income Black youth and over 20% of low-income Hispanic youth aged 14-17 years did not have home internet access in 2017. Thus, disparities in access found at earlier time points [4,35,36] have not been eliminated, and these will continue to impact youths’ ability to benefit from web-based health-related resources.

With increasing reliance on the internet for information and for a host of health and educational services, youth without home internet access are at a disadvantage [37]. Although smartphones can also be used to access the internet, and indeed many youth use smartphones, there are recognized limits to having *only* smartphone internet access [24,38]. Our finding that in 2017, 3%-5% of youth accessed the internet only from smartphones, suggests that some youth will experience challenges (eg, with connectivity and poor audio and video) [17,24,38]. The disadvantages of smartphone-only access will have a greater impact on Black and Hispanic youth living in low- and middle-income households. Similarly, research by the National Center for Educational Statistics found that 6% of children aged 3-18 years had smartphone-only access to the internet, a situation that was more common among Black, Hispanic, and low-income children [39]. In addition to challenges with connectivity, the demands of video connection (eg, telehealth visit and educational interactions) create disadvantages for those who rely only on smartphones for accessing the internet. Furthermore, even when the internet is available on a computer or smartphone, other limitations exist, such as digital literacy (eg, ability to download and install), limited internet speed that can impede using video, and incompatibility of the recipient’s devices with the platform on which the health care is being delivered [8,21,40]. Thus, although this study’s findings provide information on access and internet use in the home and via smartphone, there are additional factors that influence whether one can effectively participate in health programs or health care via the internet.

Our findings reinforce the importance of income. At both the population level and within racial and ethnic groups (ie, Black and Hispanic youth), we consistently found disparities in access for youth in low-income households. As the effects of unemployment because of the COVID-19 pandemic continue to unfold, an increasing number of households will make decisions about what is affordable. Without public and private efforts to boost internet access [41,42], more low-income households may lose this important service. Given the scope of the impact of COVID-19 on Black and Hispanic communities [43,44], gains in access between 2015 and 2017, especially among low-income households, are at risk.

Home access may have advantages over community access (eg, school and library) for youth seeking health-related information or assistance on sensitive topics (eg, sexual health and mental health; see the study by Smith-East [17]). For example, with rare exceptions, sexual content is blocked on school computers (see the study by Dolcini et al [4]), and community sites may not offer adequate privacy for health consultations [17]. Regardless of these limitations, community internet access follows similar patterns as home access, such that low-income youth have lower access than high-income youth [4]. Community closures because of COVID-19 will have further reduced access because schools, libraries, and community centers were closed for extended periods in many communities.

Although some prior research is viewed as providing evidence of nearly universal access to the internet for youth in the United States (eg, the study by Anderson and Jiang [1]), this study suggests a different reality. Our findings show continued disparities in internet access that are in alignment with other published work using population-level data (eg, ACS and the CPS) [22,23]. The perceived discrepancies in findings across studies may be explained by large margins of error for data on Black and Hispanic youth in studies that include relatively small numbers of these subpopulations. In fact, our findings with respect to household internet access for Black and Hispanic youth roughly correspond to the lower ranges of access identified by Anderson and Jiang [1] (ie, when the margin of error is taken into account). This study underscores the importance of large population-based surveys, which produce estimates with narrow CIs that avoid camouflaging population disparities. We recommend that surveys provide CIs along with point estimates to allow for cross-survey comparisons.

### Strengths and Limitations

This study has several limitations. First, the rates of internet access and use may have changed since 2017. On the basis of past trends, we anticipate that disparities by racial and ethnic groups and income would continue [4,35,36]. In addition, the 2020 recession may reverse prior gains in access. Second, we were unable to examine regional differences because the CPS was not designed to examine patterns at that level; other studies provide evidence of regional disparities in access [22]. Third, the CPS questions on internet use were updated in 2015 and one item used in this study differed between 2015 and 2017. Wording changes in items can influence responses, especially those related to smartphone use [45]. Wording changes in the item on smartphone use could have contributed to the finding showing lower use in 2017; responses to the 2015 item could have included cell phones without capacity to connect to the internet. Despite item changes, patterns observed in 2012 and in later waves of CPS were similar and broadly aligned with patterns observed in the ACS. Finally, CPS data rely on interviews with adolescents and proxies. The survey literature provides a good case for accepting proxy reports in large surveys [46-50]. Agreement between a proxy and the primary respondent is high when questions are general, observable, and use a yes/no format [49]. Fulton et al [46] demonstrated strong agreement for items in the CPS that assess computer and internet use, and laboratory studies show reliability above 90% for general items [49,50]. As noted earlier, the limitations of this study are balanced by the advantages of using population-level data sets, especially when examining racial and ethnic minority populations.

### Conclusions

Despite continued calls for universal access to the internet and the recent proposition that broadband internet access is a social determinant of health [51], this study, along with other sources [52], reveals continued disparities in access and use among youth in the United States. A variety of approaches, including private-public collaborations, increasing community *hot spots*, government sponsored expansion of broadband to rural communities, and public access broadband have potential to increase internet access of youth in the United States [37,53-55]. Along with efforts to make the internet more available to all youths to ensure access to important health resources, continued monitoring of internet access among youth using population-level data is warranted.

## Abbreviations

ACS: American Community Survey
CPS: Current Population Survey
